# Is there a correlation between upper lumbar disc herniation and multifidus muscle degeneration? A retrospective study of MRI morphology

**DOI:** 10.1186/s12891-021-03970-x

**Published:** 2021-01-19

**Authors:** Chong Liu, Jiang Xue, Jingjing Liu, Gang Ma, Abu Moro, Tuo Liang, Haopeng Zeng, Zide Zhang, Guoyong Xu, Zhaojun Lu, Xinli Zhan

**Affiliations:** 1 Spine and Osteopathy Ward, The First Affiliated Hospital of Guangxi Medical University, No.6 Shuangyong Road, 530021 Nanning, Guangxi People’s Republic of China; 2 Department of Orthopaedics, Southern Central Hospital of Yunnan Province (The First People’s Hospital of Honghe State), Mengzi, Yunnan People’s Republic of China

**Keywords:** Upper lumbar disc herniation, Multifidus muscle, Muscle degeneration, Fatty infiltration

## Abstract

**Background:**

The purpose of the study is to investigate the correlation between upper lumbar disc herniation (ULDH) and multifidus muscle degeneration via the comparison of width, the cross-sectional area and degree of fatty infiltration of the lumbar multifidus muscle.

**Methods:**

Using the axial T2-weighted images of magnetic resonance imaging as an assessment tool, we retrospectively investigated 132 patients with ULDH and 132 healthy individuals. The total muscle cross-sectional area (TMCSA) and the pure muscle cross-sectional area (PMCSA) of the multifidus muscle at the L1/2, L2/3, and L3/4 intervertebral disc levels were measured respectively, and in the meantime, the average multifidus muscle width (AMMW) and degree of fatty infiltration of bilateral multifidus muscle were evaluated. The resulting data were analyzed to determine the presence/absence of statistical significance between the study and control groups. Multivariate logistical regression analyses were used to evaluate the correlation between ULDH and multifidus degeneration.

**Results:**

The results of the analysis of the two groups showed that there were statistically significant differences (p < 0.05) between TMCSA, PMCSA, AMMW and degree of fatty infiltration. The multivariate logistic regression analysis indicated that the TMCSA, PMCSA, AMMW and the degree of fatty infiltration of multifidus muscle were correlated with ULDH, and the differences were statistically significant (P < 0.05).

**Conclusions:**

A correlation could exist between multifidus muscles degeneration and ULDH, that may be a process of mutual influence and interaction. Lumbar muscle strengthening training could prevent and improve muscle atrophy and degeneration.

## Background

Lumbar disc herniation (LDH) is a primary cause of lower back pain and sciatica in adults. Generally, in most patients the involvement of the lumbar segments L4/5 and L5/S1 are known to be associated with radicular pain, sensory deficits, or motor weakness [1]. Disc herniation at the L3/4 and above are referred to as upper lumbar disc herniation (ULDH), these account for under 10 % of lumbar disc herniations [2, 3]. The precise pathogenesis of disc herniation is yet to be established fully. However, most researchers believe it to be closely related to the anatomical characteristics [3–6].

Under normal physiological conditions, the various tissues and structures of the spine can maintain physiological stability, which prevents the spinal cord and spinal nerve roots from getting compressed or damaged [7]. The muscles that act locally to stabilize the spine include the transverse abdominis, psoas major, and multifidus muscles, and the functions of these muscles include controlling the spinal curvature and maintaining its mechanical stability. The multifidus muscles are the most important muscles for local stabilizing [8, 9]. The lumbar multifidus muscle (LMM) is the largest group of posterior muscles in the lumbosacral region [10]. The LMM specifically acts to maintain segmental stability of the spine, maintain lumbar physiological lordosis, control facet joint movement, and adjust the distribution of the intervertebral load and pressure [11]. However, unlike the other paraspinal muscles, the multifidus muscles receive only a unilateral single-segmental innervation [11, 12]. Considering the crucial roles fulfilled by the multifidus muscle for maintaining spinal stability, a decline in its physiological functions could result in changing to the original biomechanical relationship.

Recently, studies have indicated that a causal relationship may exist between LDH and multifidus muscle degeneration [13]. Compared to healthy individuals, patients with LDH have a higher degree of fatty infiltration of the multifidus muscle and smaller muscle cross-sectional area. Moreover, the associated radiological features of paravertebral soft tissue involvements, muscle degeneration and fatty infiltration were reported in LDH patients [14–17]. Reportedly, fatty infiltration of the multifidus muscle was closely related to LDH; however, such an association was not significant in adolescents [18]. Most past studies of the LMM and LDH have primarily focused on the L4/5 and L5/S1 segments [4–6, 14]. However, to the best knowledge of the authors, only a few studies have explored the relationship between multifidus muscle degeneration and ULDH. It has been demonstrated that the indirect evaluation of the features of the multifidus muscle by measuring the intensity and range of magnetic resonance imaging (MRI) signals are both effective and reliable [14, 17, 19]. This study compares the MRI features of LMM, which includes the muscle cross-sectional area, width and the degree of fatty infiltration between ULDH patients and healthy individuals, to explore the association between ULDH and the changes of the LMM.

## Methods

### Study population

Patients with single segment ULDH (herniation of the L1/2, L2/3, or L3/4) who received surgical treatment from 2012 to 2019 in the First Affiliated Hospital of Guangxi Medical University (Nanning, China) were selected as the study subjects. The diagnostic criteria are as follows: Firstly, MRI scans indicated the presence of LDH and surrounding soft tissue involvement, including the presence of compressed nerves, which constitutes a necessary diagnostic criterion. Secondly, patients with LDH present signs and symptoms consistent with the area of innervation of the affected nerve, such as radicular lower limb pain, paraesthesia, muscle weakness, diminished tendon reflexes, positive straight leg raising test/femoral stretch test, etc. It is common for LDH patients to exhibit at least three of these manifestations. Moreover, the presence of polyradiculopathies caused by a single disc herniation at higher levels requires a confirmatory diagnosis that combining clinical features and MRI. The exclusion criteria are as follows: Firstly, patients with confirmed MRI diagnosis of lower lumbar disc herniations, including L4-L5 or L5-S1 segments. Secondly, patients with other pathologies, including lumbar spinal stenosis, lumbar spondylolisthesis, scoliosis, spinal fracture, spinal tuberculosis, spinal tumours, etc. Thirdly, patients with pre-existing cardiovascular, pulmonary, cerebrovascular, and neuromuscular disorders that could lead to motor dysfunctions were excluded. Lastly, patients with a history of lumbar surgery or long-term lower back pain were also excluded. Additionally, incomplete data and poor-quality MRI images were also not included.

The control group included healthy subjects who underwent MRI examination at the outpatient department or physical examination centre of the hospital where the study was conducted, they had no history of disc herniation or lumbar degenerative diseases. The exclusion criteria include people with tuberculosis, tumours, fractures, spinal deformities, LDH, lumbar spondylolisthesis, lumbar spinal stenosis, and other lumbar spine diseases, or those with poor MR image quality that were immeasurable. The age group of all participants ranged between 20 and 65 years.

During the study period, 245 patients with ULDH were hospitalized. Under the inclusion and exclusion screening, a total of 132 patients were eligible to enter the study group (including 82 males and 50 females). The control group included healthy individuals with complete physical examination data were randomly selected for the case-control study. The collected data included age, gender, body mass index (BMI), occupation, and lumbar MRI scans for intergroup statistical comparisons. Due to random selection, there is no significant difference in the sex ratio, occupational composition and segments of the intervertebral disc between the study group and the control group.

This study was approved by the Ethics Committee of the First Affiliated Hospital of Guangxi Medical University, and obtained permissions to access clinical/personal patient data used in our research. Written informed consent was obtained from all of the participants in the study.

### Image acquisition and data analysis

**Fig. 1:**
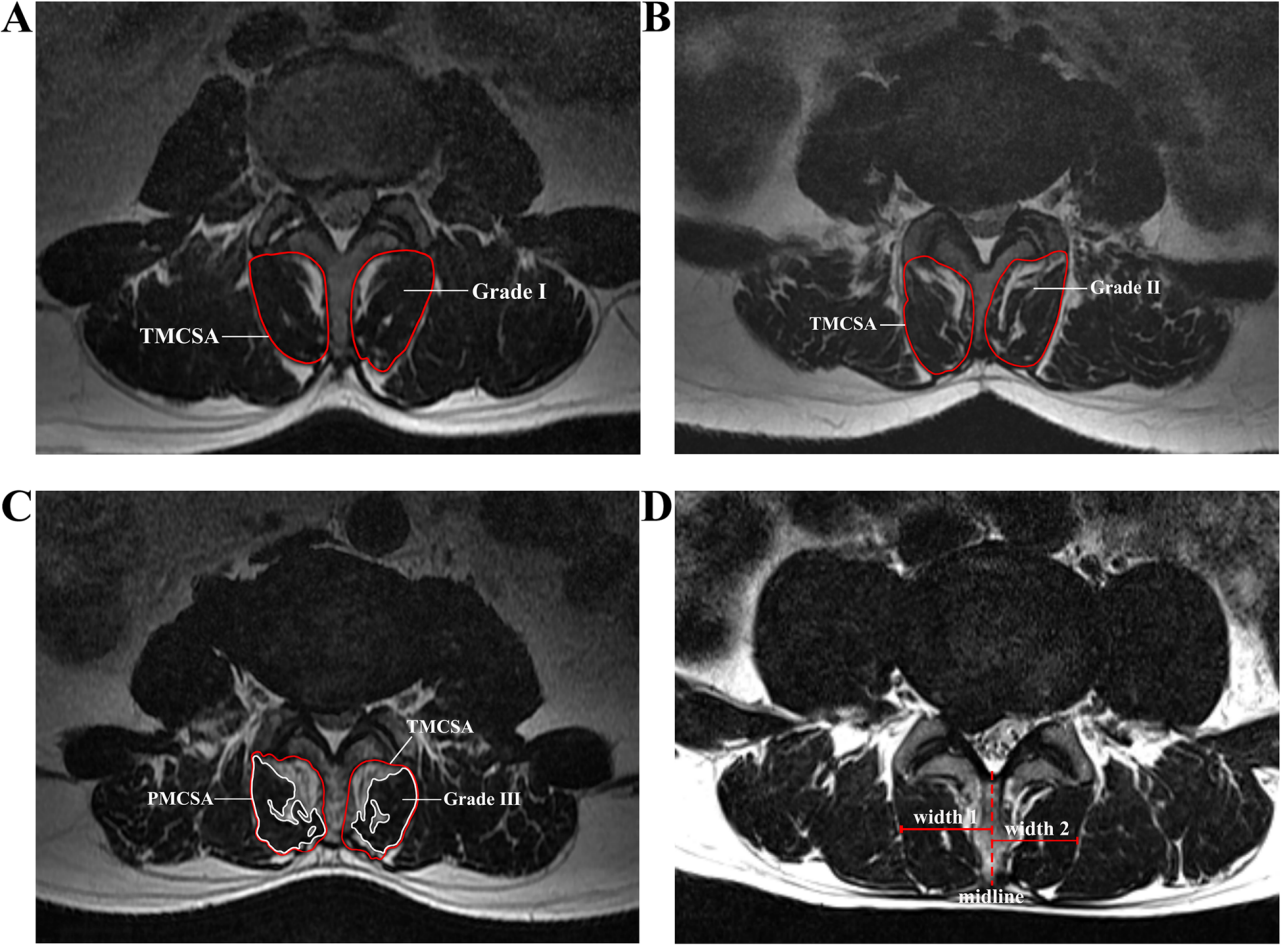
Degenerative grade of multifidus muscle, measurement range of muscle, and average width of multifidus muscle: The red-labeled circles represent the total cross-sectional area of the multifidus muscle, including muscle and interlaminar fat (**a**, **b**, **c**). The white-labeled circles represent the pure cross-sectional area of the multifidus muscle excluding interlaminar fat (**c**). The average width of the multifidus muscles on both sides is what we need (**d**). The degenerative grade of multifidus muscle: grade I (**a**), grade II (**b**), grade III (**c**). TMCSA: the total muscle cross-sectional area; PMCSA: the pure muscle cross-sectional area

The MRI data of all subjects reviewed for this study were acquired from the same MRI device (3.0 T; Magnetom Verio; Siemens Medical Solutions, Germany). And performing scans based on the following parameters: the axial T2-weighted images from L1 to S1 (TR/TE 3000/110, matrix size 256 × 256, time to recovery: 3,000–3,600 ms, time to echo: 80–105 ms, and slice thickness: 4 mm). The axial T2-weighted images of the MRI of all subjects were collected and analysed using the Picture Archiving and Communication Systems (version 2.7.1.0; PACS Clinical Viewer ANTAI; CHINA). All the obtained MRI scans of the study group were performed before the patients received surgical treatment. In the study group, a segmental plane of a herniated lumbar disc (L1/2, L2/3, or L3/4) of the axial T2-weighted images was measured at the mid-disc. The same plane of the control group was measured using data matching. All measurements were taken by a qualified radiologist with several years of experience using blinding, and measurement software. The total cross-sectional area of the muscle and the interlaminar fat were measured along the edge of the multifidus muscle (Fig. 1A, B). The PACS software was used to identify the boundary of multifidus muscle and fat in the axial images. The pure cross-sectional area of the multifidus muscle (Fig. 1C) was measured by excluding fat signal from the segmentation. The horizontal distance from the most prominent point of the outer margin of the multifidus muscle in the disc plane to the midline of the spinous process was measured to evaluate the multifidus muscle width (Fig. 1D). Based on the theoretical and practical studies of Battaglia et al.[20] and Ekin et al.[21], the visual difference between the amount of fatty infiltration was used to evaluate and grade the degree of fatty infiltration of the LMM:

Grade I: Normal muscle or mild fat infiltration, with dotted or linear infiltrated areas, fatty infiltration < 10 % (Fig. 1A).

Grade II: Moderate fat infiltration, with multiple patchy infiltrations areas, fatty infiltration 10 %-50 % (Fig. 1B).

Grade III: Severe fatty infiltration, exhibiting feathery or mesh-like infiltrations areas; fatty infiltration > = 50 % (Fig. 1C).

Statistical analyses were performed using the SPSS software (version 25.0; SPSS IBM; USA). The Chi-squared test was adopted to analyse the differences in age groupings, BMI distribution, and degree of fatty infiltration between the herniation group and the control group. The total muscle cross-sectional area (TMCSA), pure muscle cross-sectional area (PMCSA), average multifidus muscle width (AMMW), and other measurement data were measured three times, and the average of four measured values was expressed as mean ± standard deviation. The ANOVA was used to analyse and compare the differences in TMCSA, PMCSA, and AMMW between the two group. Multivariate logistic regression analysis was used to analyse the correlation between the ULDH and the factors, such as age, BMI, TMCSA, PMCSA, AMMW and the degree of fatty infiltration. *P* < 0.05 indicates the presence of statistically significant differences.

## Results

**Fig. 2:**
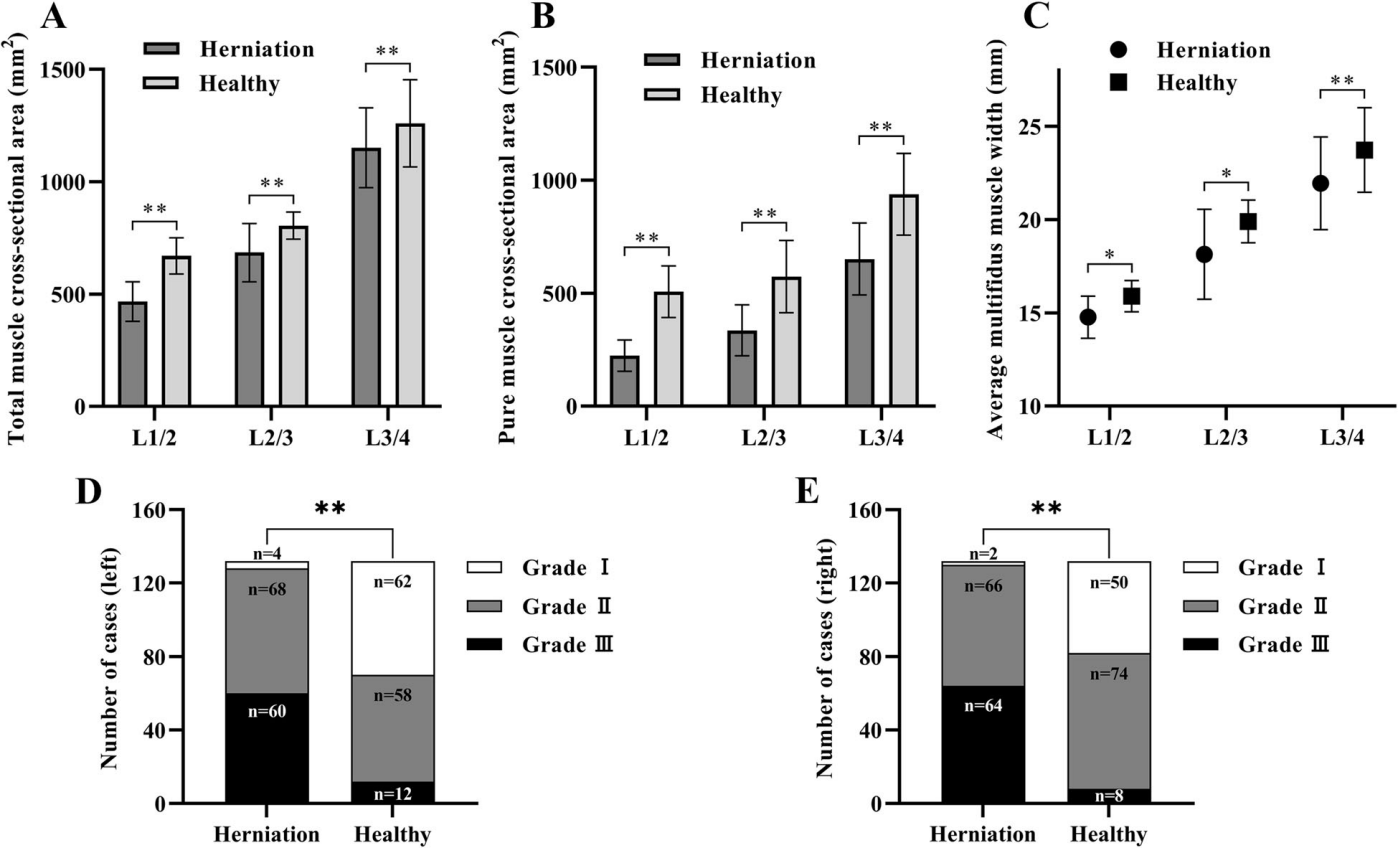
Data statistics and analysis: In the herniation and healthy groups, one-way analysis of variance applied to statistical tests. There were statistically significant differences in the total multifidus muscle cross-sectional area (**a**), pure multifidus muscle cross-sectional area (**b**) and average multifidus muscle width (**c**). Chi-square test was used to compare the degree of fatty infiltration of the left (**d**) and right (**e**) multifidus muscles in the two groups. **P<0.01. *P<0.05. LMM: lumbar multifidus muscle

This study included a total of 264 participants, including 132 in the study group and 132 cases in the control group. The distribution and characteristics of gender, profession, and the segments of the lumbar disc about the two groups were shown in Table 1. In comparison with the control group, patients with ULDH did not exhibit significant differences in age distribution and BMI distribution (*P* > 0.05). However, there were statistically significant differences (*P* < 0.05) in the TMCSA (Fig. 2A), PMCSA (Fig. 2B), AMMT (Fig. 2C), and the degree of fatty infiltration (Fig. 2D, E). The specific multifidus muscle measurement results were shown in Table 2.

**Table 1:** Characteristics of study group and control group

Feature	Study group	Control group	*P*
Age			> 0.05
Range	47.47 ± 10.918	40.27 ± 10.128	
Min - Med - Max	21-51-64	24-38.5-62	
BMI			> 0.05
< 18.5	4	10	
18.5 ~ 24.9	68	86	
≥ 25.0	60	36	
Gender			1.000
Male	82	82	
Female	50	50	
Profession			1.000
Manual workers	52	52	
Non-manual laborer	80	80	
Segments			1.000
L1/2	18	18	
L2/3	24	24	
L3/4	90	90	

*BMI *Body mass index

**Table 2:** Measurement results of the multifidus muscle

Feature	Study group	Control group	*P*
TMCSA (mm^2^)			< 0.05
L1/2	467.56 ± 87.97	670.78 ± 80.27	
L2/3	685.25 ± 129.32	804.58 ± 60.50	
L3/4	1152.11 ± 178.10	1260.91 ± 193.89	
PMCSA (mm^2^)			< 0.05
L1/2	223.89 ± 69.09	507.67 ± 114.17	
L2/3	335.92 ± 113.20	574.00 ± 159.66	
L3/4	652.36 ± 158.82	937.84 ± 181.55	
AMMW (mm)			< 0.05
L1/2	14.78 ± 1.13	15.90 ± 0.84	
L2/3	18.14 ± 2.41	19.90 ± 1.15	
L3/4	21.95 ± 2.49	23.74 ± 2.27	

*TMCSA *The total muscle cross-sectional area, *PMCSA *The pure muscle cross-sectional area, *AMMW *The average multifidus muscle width

**Fig. 3:**
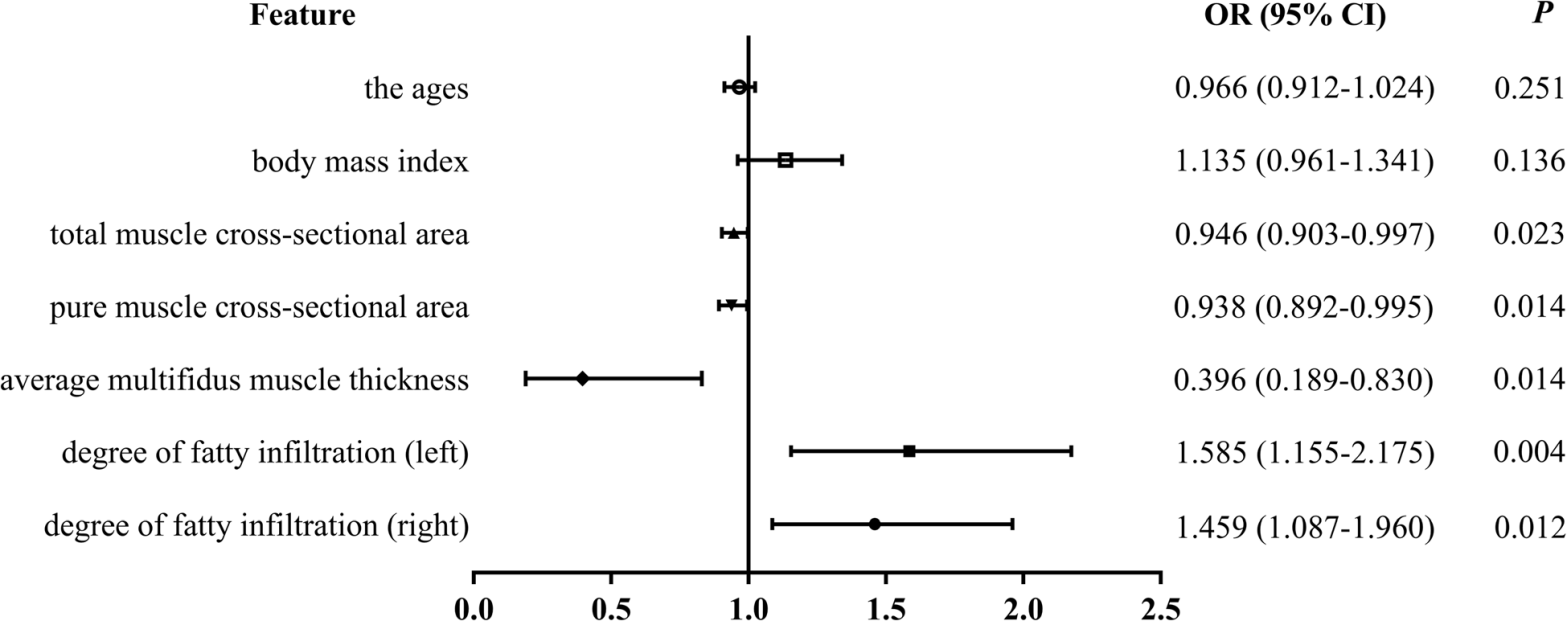
Multivariate logistic regression analysis

According to the multivariate logistic regression analysis of factors (Fig. 3), such as age, BMI, TMCSA, PMCSA, AMMW, and degree of fatty infiltration: the age (*P* = 0.251, OR 0.966, 95 %CI 0.912–1.024) and BMI(*P* = 0.136, OR 1.135, 95 %CI 0.961–1.341) were not likely to be the impact factors for ULDH (*P* > 0.05); TMCSA (*P* = 0.023, OR 0.980, 95 %CI 0.963–0.997), PMCSA (*P* = 0.014, OR 0.973, 95 %CI 0.952–0.995), AMMW (*P* = 0.014,OR 0.396, 95 %CI 0.189–0.830), degree of fatty infiltration (left: *P* = 0.004,OR 1.585, 95 %CI 1.155–2.175; right: *P* = 0.012,OR 1.459, 95 %CI 1.087–1.960) were possible impact factors for ULDH, and the differences were statistically significant (*P* < 0.05).

## Discussion

The study showed that patients with ULDH exhibited degeneration of the multifidus muscle compared with the normal control group. It was demonstrated by the decreased cross-sectional area of the multifidus muscles and increased fatty infiltration. These findings agree with those from previous studies that investigated the association between lower lumbar degeneration and multifidus muscles [14, 21].

Back pain is the most common symptom of ULDH, and the resulting release of inflammatory factors may cause multifidus muscle degeneration and a decline in function. Meanwhile, the response mechanism of the human body that restricts lumbar activity to protect itself leads to further aggravation of the degeneration of the multifidus muscle. The multivariate logistic regression analysis confirmed that the degeneration of the multifidus muscle and fatification of the multifidus muscle were the related factors of ULDH. Since this study is a retrospective study, it is difficult to establish the time sequence of the occurrence of multifidus muscle degeneration and ULDH so that the specific mechanism and relationship between both sides cannot be determined.

Studies related to the pathogenesis of multifidus muscle degeneration has revealed two main directions of study. Firstly, the analysis can consider changes to the size of muscle fibres from an anatomical and histological perspective. Kim et al. reported that in patients with LDH, the cross-sectional area of the multifidus muscle decreased significantly, meanwhile the cross-sectional area of the psoas major muscle did not show any significant changes [22]. Reportedly, in patients with LDH, the average size of the type-1 and type-2 fibres of the ipsilateral multifidus muscles was significantly smaller than that of the contralateral side. This may be related to the fact that the innervation of the multifidus muscle on the ipsilateral side was by mono-segmental nerves, thus compression or damage to these nerve roots resulted in the atrophy of the type-1 and type-2 fibres [23]. An earlier study model of porcine nerve root injury discovered that the multifidus muscle atrophied rapidly with fatty infiltration following nerve root injury. Furthermore, the study also confirmed that, degeneration of the multifidus muscle after disc disease was usually confined to a single segment [24]. Yarjanian et al. used MRI to assess the difference in the cross-sectional area of the multifidus muscle among normal subjects, patients with low back pain, and those with LDH, the authors found that the extent of multifidus atrophy could not be elucidated by denervated innervation alone, since the atrophy was reversible [25].

Secondly, the analysis is performed through the cross-sectional area and degree of fatty infiltration of the muscles from the morphological perspective. Faur et al. reported that multifidus muscle degeneration primarily occurred along the cross-section of MRI scans. Additionally, they reported that the degree of multifidus muscles degeneration was proportional to the degree of disc degeneration [26]. In this study, the patients with ULDH are significantly different from healthy people in the measurement indexes of the multifidus muscles. Moreover, the mean and range of relevant indicators are smaller. The results indicate that multifidus muscle atrophy is closely related to upper lumbar disc herniation.

Certain studies have reported that the degree of fatty infiltration of multifidus muscle at the same level as the ipsilateral segment of the LDH was significantly higher than on the contralateral segment. These findings confirmed the feasibility and reliability of visually evaluating the degree of fatty infiltration of multifidus muscle [6, 14, 17, 26]. Additionally, it was also reported that compared to assessing the cross-sectional area in patients with lumbar disc herniation, it was much more effective to assess the degree of multifidus fatty infiltration [27]. In the present study, both the cross-sectional area and the degree of fatty infiltration exhibited excellent evaluation efficiency.

Many clinical studies have confirmed the opinion that a correlation exists between multifidus muscle degeneration and LDH. However, the specific mechanism of the muscle degeneration remains unclear. Disuse and denervation are the two main mechanisms that are often mentioned [26]. Combined with the results of this study, the author assumes two models: On the one hand, degeneration of the multifidus muscles causes instability in the lumbar spine, which exacerbates degeneration of the upper lumbar intervertebral disc. On the other hand, the herniated lumbar disc presses on the nerve roots at the corresponding segment, which could lead to atrophy when the multifidus muscle is denervated.

Generally, denervation is believed to result in type-II fibre atrophy. However, there have been no unified reports on changes in type-I fibres [28]. Studies investigating the mechanism of disuse had reported a loss of functional exercise of the waist and back muscles, reduced capillary responsiveness, an insufficient supply of blood to the muscles, long-term hypoxia, and poor utilization of glycogen. The accumulation of metabolites resulted in muscle edema, and due to the disuse and degeneration of the muscles, the muscle tissues were gradually replaced by adipose tissues. It was believed that inflammatory or immunological injury mediated by inflammatory factors and injury to the psoas muscle caused by long-term heavy lumbar load degenerated the multifidus muscle [28, 29]. In an extensive study that involved 2,028 cases, it was reported that multifidus muscle degeneration was more common in adult women and the severity was directly proportional to the age [30, 31]. Reportedly, the longer the symptoms lasted, the degeneration of the multifidus muscles were more severe [32]. Lumbosacral radiculopathy, reflex inhibition of discogenic lumbago, dorsal ramus syndrome, various genetic, and environmental as well as iatrogenic factors may lead to paravertebral muscle degeneration and fatty infiltration [26, 33]. Clinical practice had often neglected the paravertebral muscles, and it was not well protected during spinal surgeries in the past. This study also confirmed that there is significant atrophy of the multifidus muscle in patients with ULDH. Future clinical practise should emphasise on the negative effects of multifidus muscle atrophy. Reinforcing the lumbar spine muscles can help improve this atrophy, which could benefit the prevention and rehabilitation of ULDH.

Therefore, detailed researches studying the association between LMM and LDH and degenerative diseases require further attention. Having a complete understanding of this relationship could help to effectively strengthening the restoration of the normal physiological shape and function, and it could also benefit the prevention and rehabilitation of LDH [34–36]. The surgical approach should be optimized to minimize damage to surrounding tissue and maintain muscular integrity and neurovascular supply [37].

This study has some limitations. Firstly, the sample size is small, which may have increased sample error. Secondly, the specific background details of the sample may require additional in-depth research to further investigate the influencing factors of multifidus muscle degeneration. This study used visual difference of MRI signal intensity of different tissues for measurement and statistics, known as the “eyeballing method”. However, this subjective visual assessment method has some limitations that cannot be ignored. Firstly, it is difficult to assess the interobserver variability. Secondly, performing drawings using the manual cursor technique could increase statistical errors. As a retrospective study, the precise mechanism and relationship between ULDH and multifidus muscle degeneration cannot be determined. Therefore, further research and analysis are needed.

## Conclusions

A relationship could exist between multifidus muscles degeneration and ULDH entailing a process of mutual influence and interaction; however, prospective studies should confirm this. Evaluation of multifidus muscles should be considered when using MRI scans to assess the ULDH patients. Lumbar muscle strengthening training could prevent and improve muscle atrophy and degeneration.

## Data Availability

The datasets used and/or analyzed during the current study are available from the corresponding author on reasonable request.

## Abbreviations

ULDH: Upper lumbar disc herniation
LDH: Lumbar disc herniation
CSA: Cross-sectional area
LMM: Lumbar multifidus muscle
MRI: Magnetic resonance imaging
PACS: Picture archiving and communication systems
TMCSA: The total muscle cross-sectional area
PMCSA: The pure muscle cross-sectional area
AMMW: The average multifidus muscle width
BMI: Body mass index
